# Journal of the International Society of Sports Nutrition: a new era begins

**DOI:** 10.1186/1550-2783-4-1

**Published:** 2007-07-13

**Authors:** Richard B Kreider

**Affiliations:** 1 Center for Exercise, Nutrition and Preventive Health Research, Department of Health, Human Performance, and Recreation, Baylor University, Waco, Texas, USA

## Editorial

The International Society of Sport Nutrition (ISSN) [1] was established in 2003 with a mission to become the leading professional organization in the field of sports nutrition. The ISSN is dedicated to promoting and supporting the science and application of sports nutrition and is recognized as the only not-for-profit academic-based society dedicated to sports nutrition and growing the science of applied nutrition. ISSN conferences, tutorials, lectures, and courses have been recognized by the American Dietetic Association (ADA), National Strength and Conditioning Association (NSCA), American College of Sports Medicine (ACSM), American Council on Exercise (ACE), American Physical Therapy Association (APTA), National Association of Athletic Trainers (NATA), and other organizations. The ISSN is recognized by many professionals in the field and Universities as offering the latest, cutting edge and non-biased information about the science of applied and practical sports nutrition.

The *Journal of the International Society of Sports Nutrition (JISSN) *is the official journal of the ISSN. The goal of the *JISSN *is to keep ISSN members, the scientific and medical community, and the general public up to date on the latest advances in exercise and sports nutrition in a well referenced but easy to understand format. In addition, to provide a quality publication outlet for exercise and sport nutrition related research. The JISSN has published reviews, original research manuscripts, case-reports, and editorials related to the role of exercise and nutrition on health, disease, rehabilitation, training, and performance.

For the first three years of publication, the *JISSN *was published as a peer-reviewed open access electronic journal accessible through the ISSN's website. While this format has been successful, the ISSN's Board of Directors [2] sought to increase the visibility, credibility, and impact of the *JISSN*. As a result, the ISSN decided to convert the *JISSN *from a society-based electronic journal to a BioMed Central journal. BioMed Central [3] is an independent publishing house committed to ensuring peer-reviewed biomedical research is open access – immediately and permanently available online without charge or any other barriers to access. Publishing the *JISSN *through BioMed Central provides an automated online submission and peer-review process; publication preparation services; the ability to publish articles online within a few days of acceptance; and, automatic indexing through PubMed [4], PubMed Central [5], Scirus [6], Google [7], Citebase [8], and OAIster [9]. Additionally, it allows for citation tracking through Thomson Scientific (ISI) [10] which will help establish and build a strong impact factor for the *JISSN*. While there is an article-processing charge [11] associated with publishing through BioMed Central, the ISSN Board of Directors believes that converting *JISSN *to a BioMed Central journal will make it more convenient for authors to submit their articles as well as greatly enhance the exposure and impact of articles published in the *JISSN*.

The *JISSN *operates a closed peer-review policy, meaning the referees' confidentiality is maintained and the reports are not made publicly available. All manuscripts submitted to the journal will be subject to immediate screening by the Editorial Board [12]. Appropriate manuscripts will be reviewed by at least two referees selected by the Editor-in-Chief, with the aim of reaching an editorial decision as soon as possible. The Editor-in-Chief is responsible for the final editorial decision. A range of article types related to the role of exercise and nutrition on health, disease, rehabilitation, training, and performance will be published. The journal provides a platform to publish exercise and nutrition related articles in an open-access manner so that readers can determine nutritional strategies that may enhance exercise and/or training adaptations leading to improved health and/or performance.

The ISSN Board of Directors, ISSN Advisory Board, and JISSN Editorial Board are very excited about the *JISSN *becoming a BioMed Central journal. We hope that exercise and nutrition researchers and practitioners will look to the *JISSN *as a quality outlet for their work. In addition, we look forward to greater exposure of the ISSN and *JISSN *within the biomedical communities and general public as we embark on a new era of publishing the *JISSN *with BioMed Central.
